# Facing Racism and Sexism in Science by Fighting Against Social Implicit Bias: A Latina and Black Woman’s Perspective

**DOI:** 10.3389/fpsyg.2021.671481

**Published:** 2021-07-16

**Authors:** Karin C. Calaza, Fátima C. S. Erthal, Mirtes G. Pereira, Kita C. D. Macario, Verônica T. Daflon, Isabel P. A. David, Helena C. Castro, Maria D. Vargas, Laura B. Martins, Jasmin B. Stariolo, Eliane Volchan, Leticia de Oliveira

**Affiliations:** ^1^Department of Neurobiology, Institute of Biology, Universidade Federal Fluminense, Niterói, Brazil; ^2^Laboratory of Neurobiology, Instituto de Biofísica Carlos Chagas Filho, Universidade Federal do Rio de Janeiro, Rio de Janeiro, Brazil; ^3^Department of Physiology and Pharmacology, Biomedical Institute, Universidade Federal Fluminense, Niterói, Brazil; ^4^Department of Physics, Institute of Physics, Universidade Federal Fluminense, Niterói, Brazil; ^5^Department of Sociology and Methodology of Social Sciences, Institute of Human Sciences and Philosophy, Universidade Federal Fluminense, Niterói, Brazil; ^6^Department of Cellular and Molecular Biology, Institute of Biology, Universidade Federal Fluminense, Niterói, Brazil; ^7^Chemistry Institute, Universidade Federal Fluminense, Niterói, Brazil; ^8^Biomedical Institute, Universidade Federal Fluminense, Niterói, Brazil; ^9^Institute of Biology, Universidade Federal Fluminense, Niterói, Brazil

**Keywords:** implicit bias, stereotype threat, gender inequalities, diversity, underrepresented groups

## Abstract

The editors of several major journals have recently asserted the importance of combating racism and sexism in science. This is especially relevant now, as the COVID-19 pandemic may have led to a widening of the gender and racial/ethnicity gaps. Implicit bias is a crucial component in this fight. Negative stereotypes that are socially constructed in a given culture are frequently associated with implicit bias (which is unconscious or not perceived). In the present article, we point to scientific evidence that shows the presence of implicit bias in the academic community, contributing to strongly damaging unconscious evaluations and judgments of individuals or groups. Additionally, we suggest several actions aimed at (1) editors and reviewers of scientific journals (2) people in positions of power within funding agencies and research institutions, and (3) members of selection committees to mitigate this effect. These recommendations are based on the experience of a group of Latinx American scientists comprising Black and Latina women, teachers, and undergraduate students who participate in women in science working group at universities in the state of Rio de Janeiro, Brazil. With this article, we hope to contribute to reflections, actions, and the development of institutional policies that enable and consolidate diversity in science and reduce disparities based on gender and race/ethnicity.

## Introduction

“Science has a racism problem,” claimed an editorial of the important journal “Cell” (Edge, 2020). Editors from a variety of respected scientific journals, such as Nature and Science, have recently asserted the importance of combating racism and sexism in science. Especially after the COVID-19 pandemic, several pieces of evidence suggest that gender and racial gaps may be widened (Collins et al., 2020; Myers et al., 2020; Staniscuaski et al., 2020). For instance, Staniscuaski et al. (2021), analyzing academic productivity, showed that male academics—especially childless academics—were the group least affected by the pandemic. In contrast, female academics, especially Black women and mothers, were the most impacted group.

Although the fight against racism and sexism in science involves several aspects, socially constructed implicit bias is a key component in this fight. “Bias” is a concept that refers to analysis, judgments, or attitudes that do not adhere to the principles of impartiality. Bias against a person or group can lead to unfair assessments. This judgmental bias can be explicit or implicit (not perceived), and it can occur due to skin color, ethnicity, religion, gender, sexual orientation, weight, physical, or mental disability, among others (Greenwald and Krieger, 2006; Staats et al., 2015). Implicit (unconscious or unperceived) negative judgment bias in the academic sphere is generally associated with social stereotypes of individuals who are stigmatized as intellectually limited or incapable. Importantly, a social stereotype is a mental association of a social group or category with a characteristic or trait that may or may not be favorable (Greenwald and Krieger, 2006). In other words, stereotypes are socially constructed beliefs that do not necessarily reflect reality (Allport, 1954; Ashmore and DelBoca, 1981; Greenwald and Banaji, 1995). Such social constructions, which are determined by culture and the unequal distribution of resources and power in a community, have substantial influence on the unconscious evaluations and judgments of individuals or groups (Staats et al., 2015; Storage et al., 2016). Stereotypes that are repeatedly and imperceptibly transmitted through several information channels induce implicit beliefs that are used to organize and socially categorize the world and provide rationales for entrenched inequalities (Gaucher et al., 2011; Kang, 2012; Gálvez et al., 2019; Rivera and Tilcsik, 2019). These implicit associations are more prevalent than explicit prejudice, which means that even people who consciously believe in and defend the principles of justice and non-discrimination can have their judgment affected by implicit bias, without their knowledge (Staats et al., 2014). In fact, evidence suggests that implicit bias can be a better predictor of behavior than explicit bias (Bargh and Chartrand, 1999; Ziegert and Hanges, 2005). While explicit biases are conscious attributions that are accessible through introspection, implicit biases are more difficult to become conscious of. Nevertheless, implicit bias can be assessed through experimental paradigms using a diversity of approaches and research tools (see below).

## Implicit Gender Bias

Negative implicit stereotypes are shaped by experience and are based on implicit learned associations between the culturally constructed putative characteristics of members of social categorical groups, including those based on race, gender, and socioeconomic status. The presence of these stereotypes leads to implicit bias in judgments of stigmatized individuals or groups (Greenwald and Banaji, 1995). The formation of implicit gender stereotypes, which associate characteristics of exceptional brilliance and intelligence to the male gender, seems to start early in life (Bian et al., 2017) and is reinforced by daily experiences in which members of a categorical group appear to be associated with economic precariousness and a lack of power (Tilly, 1998). In the study of Bian et al. (2017), children from 5 to 7 years old listened to a text that described a brilliant person. Then, children viewed pictures of women’s and men’s faces and were asked to indicate which person was the character in the story. Among the five-year-old children, both boys and girls chose photographs of people of their own gender. However, among children aged 6 and older, only boys continued to indicate the pictures of people of their own gender as the brilliant character in the story, while girls became less likely to choose photographs of women. Considering that children at this age generally show positive biases toward their own in-groups (e.g., those of the same gender), this result suggests that the consequences of the stereotype that brilliance is a male characteristic occur very early and that this stereotype already begins to impact girls between 5 and 6 years old (Bian et al., 2017). Interestingly, a study showed that national gender differences in science and math success are associated with national differences in implicit gender-science stereotypes. Specifically, the stronger the nation’s citizens’ implicit association of men with science and women with the liberal arts, the greater the gap between female and male adolescents’ eighth-grade science achievement in that nation (Nosek et al., 2009). There is evidence that implicit bias acts incisively in adulthood, harming women. One study showed that when university faculty (both men and women) analyzed an identical curriculum for a laboratory manager position with either a male or a female name, the faculties evaluated the curriculum with a male name as more competent and deserving a higher salary (Moss-Racusin et al., 2012). In the same vein, Reuben et al. (2014) carried out a study in which participants (men and women) who were volunteers in laboratory research were rewarded for “hiring” a good candidate to perform mathematical tests. Women were systematically less chosen than men in all three experimental conditions tested as: (1) a condition in which no skill information and only information about the physical appearance of the candidates was provided (2) a condition in which the candidates could give a speech to talk about their mathematical skills, and (3) a condition in which information about the candidates’ performance on a previous math test was provided. Interestingly, in this last experimental condition, the power of the effect of implicit bias was clearly demonstrated, as the “employers” preferred to choose men with low performance in mathematics over women with good performance. The authors also reported that in condition (2), when the candidates were allowed to talk about their skills, the male candidates overestimated their math skills, while the female candidates did the opposite.

The presence of this implicit bias against women causes considerable damage to the development of their scientific careers. Only 18.1% of articles published in high-impact journals (Nature research journals) have women as senior authors (last authorship), and the higher the journal’s impact index is the smaller the number of women listed as the principal author (Bendels et al., 2018). In addition, articles with women as the principal author are less cited than those with men as the principal author (Larivière et al., 2013). Recently, Dworkin et al. (2020) analyzed high-impact neuroscience journals and found that papers with men listed as the first or last author were cited 11.6% more than expected given the proportion of such articles in the field, and papers with women listed as the first or last author were cited 30.2% less than expected. Importantly, however, when articles are reviewed anonymously (double-blind review), the number of articles published with women listed as the first author increases (Budden et al., 2008), highlighting the impact of implicit bias in this process. Women who have authored the same number of publications with the same publication impact as men are less likely to become research leaders (Van Dijk et al., 2014). Additionally, letters of recommendation written for women use significantly fewer adjectives that represent intelligence and brilliance (Dutt et al., 2016; Kuo, 2016).

In terms of research funding, the effects of implicit bias against women are also significant. A study based on data from a Swedish funding agency reported that women need to author twice as many publications to obtain the same scientific competence score as men (Wenneras and Wold, 1997). Recently, a study based on funding provided by the NIH (a US research funding agency and one of the largest such agencies in the world) revealed that men obtain more funding renewal than women (Pohlhaus et al., 2011). A Dutch study showed no difference between men and women in the quality of the research proposal/project submitted for funding. However, in their sample, women received less funding due to lower scores in the “quality of the researcher” (Van der Lee and Ellemers, 2015). In the same vein, a Canadian study showed that the funding gap is generated by an unfavorable view of women as scientific leaders and not based on the quality of their studies (Witteman et al., 2019). Importantly, when evaluation committees of funding agencies are aware of gender bias against women, the unequal distribution of funding between men and women is less likely to occur (Régner et al., 2019).

## Implicit Racial/Ethnicity Bias

Although the studies discussed above focus on gender stereotypes, the literature also describes implicit judgment bias based on skin color and ethnicity. For example, in one study, fictitious resumes with white-sounding names received 50% more callbacks for interviews than resumes with African-American-sounding names (Bertrand and Mullainathan, 2004). Jaxon et al. (2019) demonstrated in children that the association of brilliance with male gender might depend on the race of the person being evaluated. This intersectional study showed that children associated brilliance with White men but not with Black men (Jaxon et al., 2019). Storage et al. (2016) evaluated the frequency with which college students commented whether their professors were “brilliant” or a “genius” in course reviews on a popular Web site.^1^ They showed that fields in which “brilliant” and “genius” appeared more often were also less likely to be pursued by African–American PhDs, predicting less diversity at the PhD level. This evidence indicates a strong racial bias that helps explain, for instance, the extremely low percentage of faculty positions and PhDs earned by African Americans in STEM (National Science Foundation, 2015; U.S. Department of Education, 2017; Bernard and Cooperdock, 2018). Baron et al. (2006) used an adaptation of the implicit association test (IAT; Greenwald et al., 1998) to assess racial bias in children. Reaction time paradigms, on which the IAT is based, have been long used in studies of attention and motivation. Faster or slower response can indicate preset congruent or incongruent association in brain processing, respectively. Baron et al. (2006) tested for associations between the stereotyped group (race: Black and White) and stereotyped domain (evaluation: words with positive connotations and words with negative connotations) and showed that negative implicit race bias was already present in white children aged 6–10 years. The authors also observed that explicit beliefs about race became more egalitarian over time, but implicit race bias remained unchanged.

In a very recent interesting study, Eaton et al. (2020) probed the implicit bias for gender and its association with race/ethnicity. The authors developed an experimental design in which physics and biology professors from United States Research Universities were asked to evaluate identical curriculum vitae (CV) depicting a hypothetical doctoral graduate applying for a postdoctoral position in their field. The reviewers were asked to rate the candidate on competence, hireability, and likeability. The candidate’s name on the CV was used to manipulate race/ethnicity (Asian, Black, Latinx, and White) and gender (female or male), with all other aspects of the CV being the same across conditions. The authors found for physics reviewers an interaction between candidate gender and race/ethnicity. Black women and Latinx candidates were rated the lowest in hireability. This result suggested the robust combined effect of gender and racial/ethnicity biases.

The stereotype of being incompetent/unreliable (Fiske et al., 1999; Jimeno-Ingrum et al., 2009; Pérez, 2010) creates unfair disadvantages for Latinx scientists, especially in the context of leadership roles or to gain recognition for their studies. The persistent lack of Latinx and African representation on editorial boards is an example of the consequences of racism in the academic world (Espin et al., 2017). Latinx exclusion is so problematic that even the widely applied test used to detect/study automatic attitudes and implicit bias for putative stereotype groups, IAT, did not originally include this topic. The first study to adapt an IAT to detect implicit bias toward Latinx individuals was developed much later than the original studies (Pérez, 2010). Thus, discussions about implicit bias and stereotypes and their harmful effects are imperative in science and should consider the intersections between gender and race/ethnicity.

## Stereotype Threat

Another harmful consequence of unfounded cultural stigma is low performance on cognitive tasks generated by the threat of stereotypes. Stereotype threat is a psychological phenomenon that involves people feeling at risk of conforming to negative stereotypes about their social group (Steele and Aronson, 1995; see also the review by Spencer et al., 2016). Stereotype threat makes an individual feel a sense of exclusion and lack of belonging that generates psychological stress or anxiety and impairs performance in different situations. Social bonds are necessary for survival and are extremely salient in human beings (Tomasello, 2014), which was highlighted by the COVID-19 pandemic (Bzdok and Dunbar, 2020). Human beings have a constant motivation to form and maintain lasting, positive, and significant interpersonal relationships, even in only a minimal number of these relationships (Baumeister and Leary, 1995). Likewise, perceived social isolation is one of the most pervasive threats to human wellbeing (Cacioppo and Cacioppo, 2014). Humans react to cues of social rejection or exclusion by triggering the autonomic, endocrine and immune systems similarly to when confronting physical attacks or life-threatening events (Eisenberger, 2012), leading authors to tie the word “pain” to both physical and social wounds (see Eisenberger et al., 2003). In fact, neuroimaging studies have shown an overlap of neural representations for social and physical pain (Kross et al., 2011; Eisenberger, 2012). Indeed, in the most efficient experimental protocol to study stress, participants perform speech and cognitive tasks while being ostensively evaluated by a board of trained researchers (Kudielka et al., 2007). The potentially negative evaluation and the fear of failure trigger the reactions of social pain, focusing attentional resources on the threat and weakening performance (Gruenewald et al., 2004; Angelidis et al., 2019).

Belonging to a group stigmatized by negative stereotypes in academic domains exacerbates the pain of social isolation, causing an upward spiral of physiological and mental stress and harmful impairments to performance (Blascovich et al., 2001; Croizet et al., 2004; Allen and Friedman, 2015). Stereotype threat also reduces working memory capacity (Schmader and Johns, 2003; Rydell et al., 2009), which is extremely important to perform well in tasks. Working memory is diverted to address the survival-related threat of social exclusion through intrusive thoughts, anxiety, and stress that are imposed by stereotype threat (Schmader and Johns, 2003). Thus, unsurprisingly, even subtle situational cues for the stress due to stereotype threat can lead to a reduction in performance. In the seminal studies by Steele and Aronson (1995), the authors showed that African American college students performed worse than European American college students on a verbal task under an experimental condition of stereotype threat, in which the task was described as a “diagnostic of intellectual ability.” In the non-stereotype threat condition, in which the task was described as “a laboratory problem-solving task that was non-diagnostic of ability,” Black and white participants performed equally (Steele and Aronson, 1995). Employing a similar paradigm in France, Croizet and Claire (1998) showed that students with low socioeconomic status performed significantly worse than those with high socioeconomic status in the diagnostic condition but equally well in the non-diagnostic condition. Désert et al., 2009 observed that children with low socioeconomic status (6–9 years old) are already vulnerable to stereotype threat. Low-status children performed significantly worse under a diagnostic condition than under a non-diagnostic condition in a test of intellectual ability, whereas high-status children were unaffected. Other experimental approaches showed undermining of women’s performance in mathematical tests by inducing subtle cues of gender stereotype threat (e.g., Spencer et al., 1999; Dar-Nimrod and Heine, 2006). Indeed, math-gender cultural stereotypes seem to already affect girls, both implicitly and explicitly, at 6–10 years old (Cvencek et al., 2011).

Furthermore, Johns et al. (2005) performed a study in which men and women completed difficult math problems that were described as a problem-solving task “for a study of general aspects of cognitive processes” or a math test “for a study of gender differences in mathematics performance.” As expected, the results showed that women performed worse than men when the problems were described as a math test because of the stereotype threat created by the association between women and poor performance in math. Interestingly, when the participants were informed about the stereotype threat phenomenon, the differences in performance between women and men disappeared, indicating that “knowing is half the battle,” as the authors suggested in the paper title. Despite all the evidence showing that the stereotype threat is a robust phenomenon, some experimental paradigms have failed to replicate these data or generalize from the laboratory to real-world testing situations (Cullen et al., 2004, 2006; Sackett et al., 2004). However, as pointed out by Spencer et al. (2016), there is converging evidence that indicates that the stereotype threat is, in fact, responsible for decreases in performance in real tests. In addition, as suggested by Spencer et al. (2016), the experimental design must be carefully planned to capture the phenomenon of stereotype threat.

Considering these data, individual and institutional actions to disseminate this knowledge about stereotype threat are fundamental to reduce it among stereotyped groups. We believe these actions would be a powerful approach to fight racism, gender disparity, and the false belief of low intellectual ability of those from disadvantaged socioeconomic environments.

**Figure 1:**
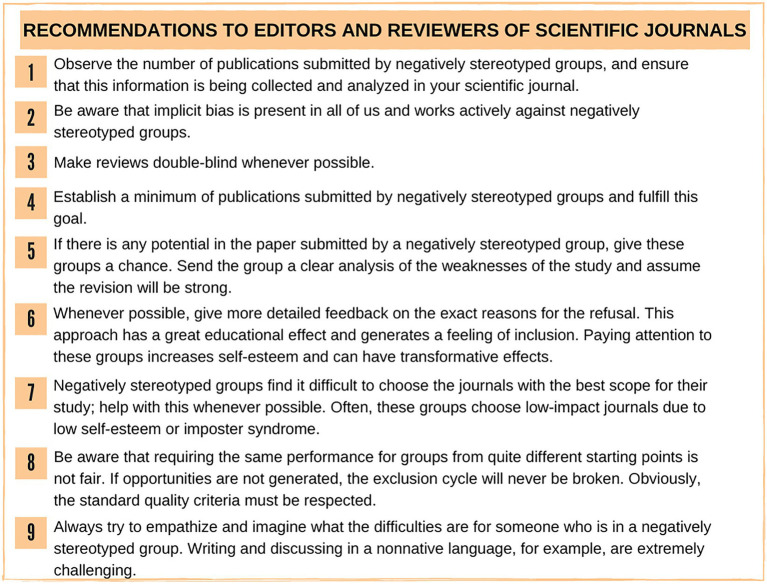
Suggestions for people in positions of power within scientific journals.

**Figure 2:**
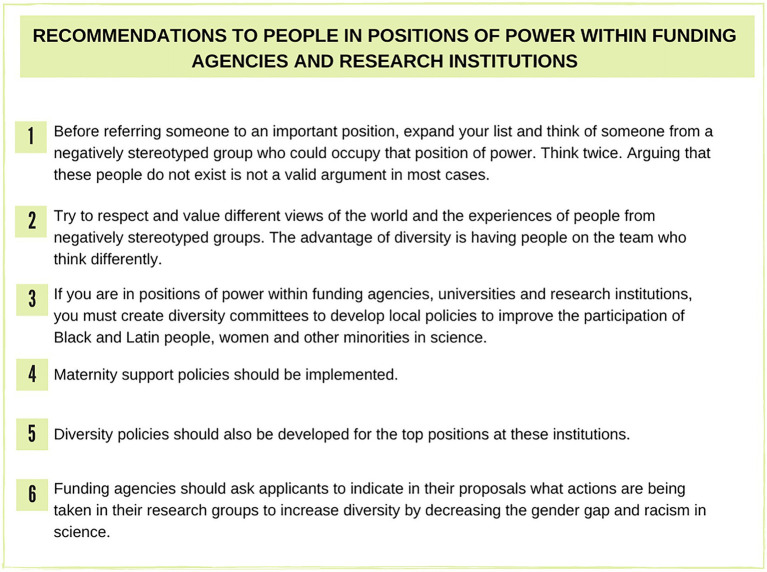
Suggestions for people in positions of power within funding agencies and research institutions.

**Figure 3:**
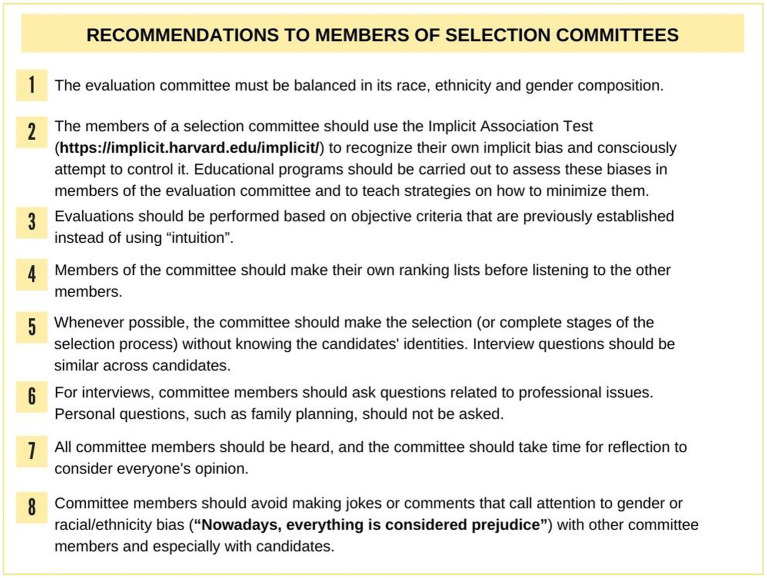
Suggestions for people in positions of power within selection committees.

In sum, there is ample evidence indicating the presence of unseen forces that work to prevent the progression of women, Latinx, and Black people to positions of greater prominence and leadership, including in the academic world. In Figures 1–3, we suggest several actions aimed at (1) editors and reviewers of scientific journals (2) people in positions of power within funding agencies and research institutions, and (3) to members of selection committees to mitigate this effect. These recommendations are based on the experience of a group of Latinx American scientists comprising Black and Latinx women, teachers, and undergraduate students who participate in women in science working group at universities in the state of Rio de Janeiro, Brazil.

## Why is Diversity Important for Science?

Diversity in science can promote new discoveries, as it expands the points of view, issues, and areas addressed by researchers (Nielsen et al., 2017). Scientists from different backgrounds may choose to investigate different questions, and more importantly, they may approach the same question in different ways. For instance, historically, bird song has been associated with males seeking to attract females. However, a deeper look at this question performed by women researchers showed that female song is common and that both sexes probably sang in the common ancestor of modern songbirds (Riebel et al., 2019). Hong and Page (2004) showed that when participants try to solve complex problems, the ability to see the problem differently, not simply “being smart,” often is the key to discovery. Indeed, when groups of different individuals are working to solve difficult problems, the diversity of the problem-solvers matters more than their individual ability. Another important example of the importance of diversity in the coordination of scientific research concerns the understanding of physiological differences related to health problems. There is evidence that diversity among doctors and health professionals improves access to care for underprivileged groups, develops culturally informed care, and expands the health research agenda (Cohen et al., 2002; Jackson and Gracia, 2014; Valantine and Collins, 2015). Then, diversity promotes perspectives from different angles, contributing to a more complete understanding of the topic.

Despite the importance of diversity in science, research conducted by underrepresented groups is frequently underestimated. Hofstra et al. (2020) showed that underrepresented groups produce higher rates of scientific novelty. Surprisingly, this study showed that the innovative and disruptive contributions made by underrepresented groups are undervalued and are less accepted by other scholars than are new contributions by gender and racial majorities. In addition, they showed that equally impactful contributions from gender and racial minorities are less likely to result in successful scientific careers. This evidence shows the inequality and injustice that is perpetuated in science. For the building of a fair and truly excellent scientific community, we need efficient policies that promote gender and racial/ethnicity equity.

## Conclusion

Converging evidence in the literature suggests that explicit and implicit biases related to gender and race/ethnicity are powerful forces that foster the disparities and inequalities found in our society. Cognitive control can allow individuals to more easily refute explicit bias as they consciously perceive it. However, implicit bias is more prevalent than explicit bias. Therefore, it is crucial to increase awareness of the commonly ignored implicit biases so that each of us can cognitively resignify them. Additionally, institutions must submit proposals to mitigate this problem. With this article, we hope to contribute to reflections, actions, and the development of institutional policies that enable and consolidate diversity in science and reduce disparities in gender, race/ethnicity, which is essential to improve innovation and, therefore, the progress of inclusive science. If we want to combat racism and sexism in science, we need to combat socially constructed implicit bias. This issue is especially important now, as the COVID-19 pandemic may widen the gender and racial gap. Implicit bias is an unseen force that prevents us from moving toward the construction of a more inclusive and diverse science.

## Author Contributions

KC, LO, FE, and EV conceived the presented idea of writing a manuscript on this subject. All authors contributed to the literature review and discussion of recommendations. All authors contributed to the final version of the manuscript.

### Conflict of Interest

The authors declare that the research was conducted in the absence of any commercial or financial relationships that could be construed as a potential conflict of interest.

## Footnotes

^1^http://RateMyProfessors.com
